# Reviewer acknowledgement 2015

**DOI:** 10.1186/s13100-015-0036-6

**Published:** 2015-03-26

**Authors:** Nancy L Craig, Thomas H Eikbush, Cédric Feschotte, Henry Levin

**Affiliations:** Johns Hopkins School of Medicine, 725 N. Wolfe Street, 502 PCTB, Baltimore, MD 21205 USA; University of Rochester, Department of Biology, River Campus Box 270211, Rochester, NY 14627-0211 USA; Department of Human Genetics, University of Utah School of Medicine, Salt Lake City, UT USA; Eunice Kennedy Shriver National Institute of Child Health and Human Development, National Institutes of Health, Bethesda, MD USA

## Abstract

The Editors of *Mobile DNA* would like to thank all our reviewers who have contributed to the journal in volume 5 (2014).

**Wenfeng An**

United States of America

**Irina Arkhipova**

United States of America

**Peter Atkinson**

United States of America

**Daniel Barbash**

United States of America

**Marlene Belfort**

United States of America

**Guillaume Bourque**

Canada

**Frederic Brunet**

France

**Kathleen Burns**

United States of America

**Pierre Capy**

France

**Richard Cawthon**

United States of America

**Wayne Decatur**

United States of America

**Prescott Deininger**

United States of America

**Keith Derbyshire**

United States of America

**David Edgell**

Canada

**Anthony Furano**

United States of America

**Jose Luis Garcia-Perez**

Spain

**Clement Gilbert**

France

**Valer Gotea**

United States of America

**Ning Jiang**

United States of America

**I. King Jordan**

United States of America

**Aurelie Kapusta**

United States of America

**Haig Kazazian**

United States of America

**Dusan Kordis**

Slovenia

**Zev Kronenberg**

United States of America

**Ashwini Kucknoor**

United States of America

**Emmanuelle Lerat**

France

**Elgion Loreto**

Brazil

**Harmit Malik**

United States of America

**Colin Malone**

United States of America

**Francois Michel**

France

**Olivier Panaud**

France

**Joseph Peters**

United States of America

**Ramesh Pillai**

France

**Ellen Pritham**

United States of America

**David Ray**

United States of America

**Francois Sabot**

France

**Gerald Schumann**

Germany

**Haruhiko Siomi**

Japan

**Jainy Thomas**

United States of America

**Chantal Vaury**

France

**Cristina Vieira**

France

**Thomas Wicker**

Switzerland

**David Witherspoon**

United States of America

**Steven Zimmerly**

Canada

